# Treatment of fungal myositis with intra-lesional and intravenous itraconazole: a case report

**DOI:** 10.1186/1752-1947-7-132

**Published:** 2013-05-19

**Authors:** Xiao-Ji Lin, Rong-Xin Yao, Mu-Qing He, Bao-Ling Zhu, Wen-Jian Guo

**Affiliations:** 1 Department of Haematology, The Second Affiliated Hospital of Wenzhou Medical College, Zhejiang, China

**Keywords:** *Candida krusei*, Fungal myositis, Intra-lesional itraconazole

## Abstract

**Introduction:**

Fungal myositis is very uncommon, even in patients who are immunocompromised. Because of its rarity and a lack of clinical experience, no consensus has been reached about the best means of treating fungal myositis. To the best of our knowledge this is the first description of the treatment of fungal myositis with simultaneous intravenous and intra-lesional itraconazole.

**Case presentation:**

A 35-year-old Chinese woman with acute myelomonocytic leukemia developed *Candida krusei* fungemia and fungal myositis in the right biceps brachii after chemotherapy. A course of intravenous itraconazole and subsequently intravenous voriconazole was initiated and her blood cultures became sterile; however, our patient remained febrile and the myositis did not resolve. Intravenous itraconazole was restarted simultaneously with low-dose intra-lesional itraconazole. The pyrexia settled after 48 hours and within 10 days the lesion could be seen to be resolving. After the course of intravenous and intra-lesional anti-fungals was complete, oral itraconazole was administered as maintenance therapy.

**Conclusions:**

To the best of our knowledge this is the first case in which fungal myositis was successfully treated with intravenous and intra-lesional itraconazole in a patient with acute myelomonocytic leukemia. The efficacy and safety of locally-administered itraconazole to treat intractable soft tissue infections requires further evaluation.

## Introduction

There has been a steady increase in the frequency of opportunistic invasive fungal infections (IFIs) in patients who are immunocompromised, mostly affecting the broncho-pulmonary tract [1]. Even so, fungal myositis, an infection of skeletal muscle, is rare. Regardless, several cases of fungal myositis caused by *Candida*, *Aspergillus* and *Cryptococcus* have been reported in patients who are immunocompromised [2-5]. As it is rare, individual clinicians have rarely seen more than a handful of cases and therefore no evidence-based treatment guidelines have been established beyond the prompt systemic administration of effective anti-fungal agents. We report a case in which fungal myositis was treated effectively with intra-lesional and intravenous itraconazole in a woman with acute myelomonocytic leukemia.

## Case presentation

A 35-year-old, 52kg Chinese woman with acute myelomonocytic leukemia (M_4_) was admitted to our hospital with fever. Our patient had been diagnosed as having leukemia seven months previously and had received only three cycles of standard induction chemotherapy (cytarabine 100mg/m^2^ on days one to seven and with idarubicin 10mg/m^2^ on days one to three) due to difficulty in paying for the treatment. At the time of her admission she was initially treated with broad-spectrum antibiotics (cefoperazone-sulbactam and levofloxacin).

**Figure 1:**
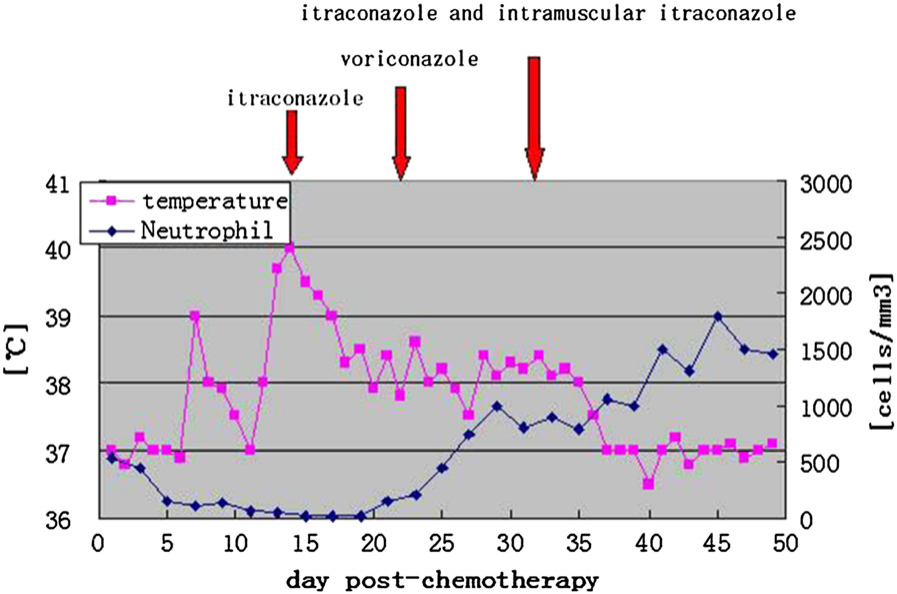
Thermometric evolution and anti-fungal drug therapy.

**Figure 2:**
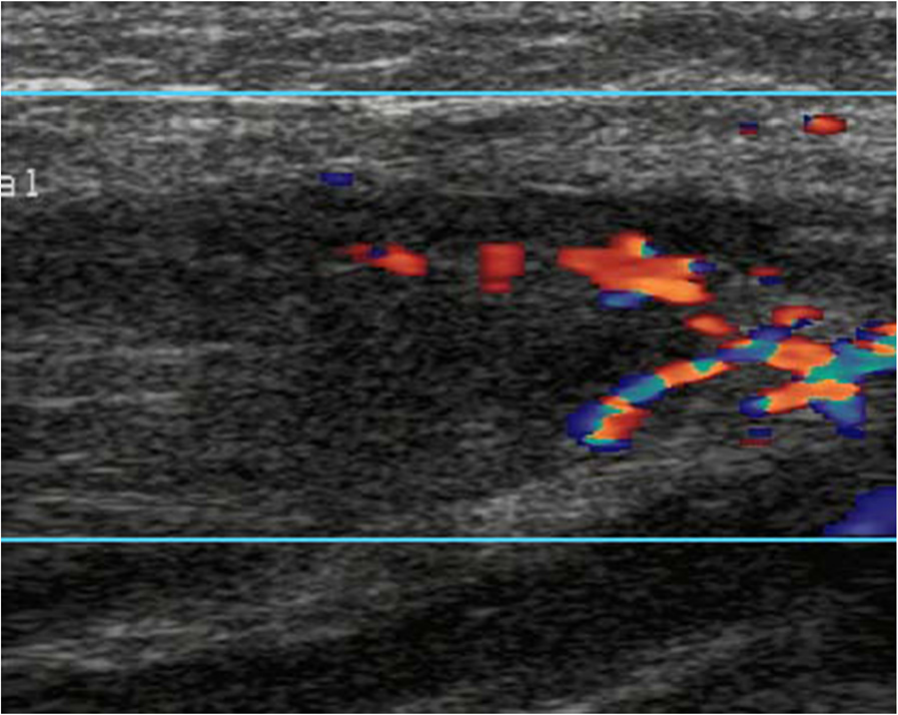
Ultrasonography of the right biceps brachii showed a hypo-echoic lesion within the muscle in which blood supply could be detected by color Doppler flow imaging.

**Figure 3:**
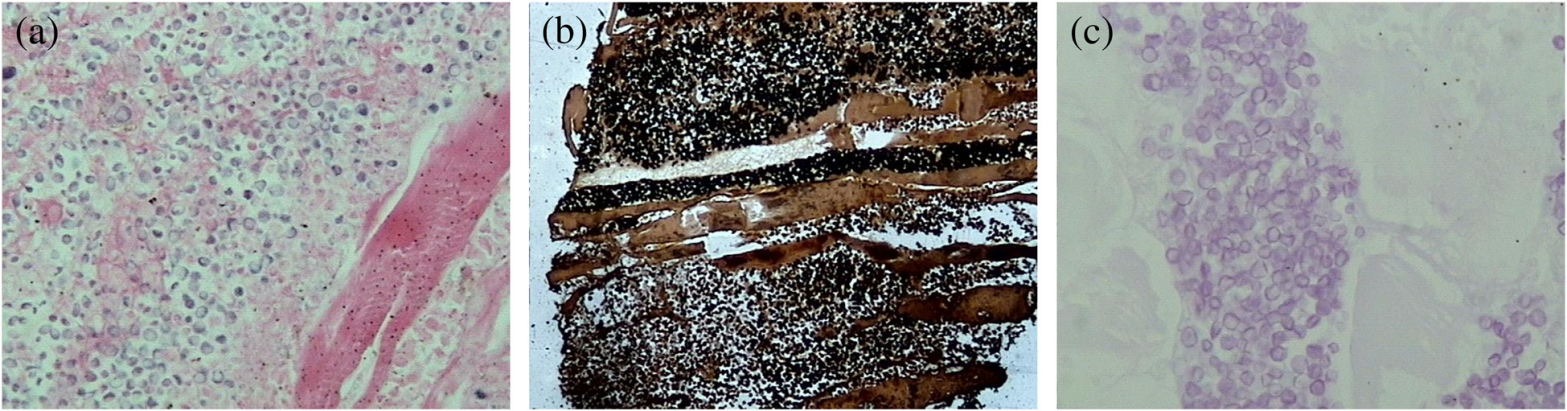
**A histopathological analysis of muscle tissue showed focal necrosis with inflammatory cell infiltration and diffuse fungal spores among the muscle cells. (a)** hematoxylin and eosin stain, ×100; **(b)** Grocott’s methenamine silver stain, ×100; **(c)** periodic acid-Schiff stain, ×400.

The results of a physical examination revealed superficial multiple lymphadenectasis, and our patient’s general condition was good. A full blood count revealed a neutrophil count of 940 cells/mm^3^. Urine analysis, a computed tomography (CT) scan of the chest and abdominal ultrasonography results were normal. Blood, urine and stool cultures were also negative. Bone marrow cytomorphology showed that monoblasts and promonocytes comprised 48.6 percent of non-erythroid cells. Subsequently, our patient received fludarabine, cytarabine, and granulocyte-macrophage colony-stimulating factor (FLAG) chemotherapy (cytarabine 1g/m^2^ on days one to five, with fludarabine 25mg/m^2^ also on days one to five). After five days of chemotherapy, the neutrophil count had fallen to 150 cells/mm^3^ so she was treated with granulocyte-macrophage colony-stimulating factor. At day 7, high pyrexia was evident, but the results of a repeat chest CT scan was normal and blood cultures were sterile. Empiric therapy with imipenem/cilastatin and vancomycin was started. On day 13, high pyrexia recurred and subsequently two separate peripheral venous blood specimens grew *Candida krusei*, which was sensitive to itraconazole, voriconazole and amphotericin B, but resistant to fluconazole and 5-fluorouracil. Intravenous itraconazole (400mg loading dose for the first two days followed by 200mg daily) was started (Figure 1). On the 15th day our patient reported right upper arm pain with signs of erythema, swelling and hyperalgesia; ultrasonography revealed a hypo-echoic lesion within the right biceps brachii, 19×55mm in size, in which blood flow could be detected by color Doppler flow imaging (Figure 2). By day 17, her temperature had gone down to 38.0 to 38.5°C, but at day 25 a low grade fever persisted and the biceps lesion had not changed, although the blood cultures no longer grew *Candida*. Histopathological examination of a biopsy specimen from the lesion showed dense fungal spores, no hyphae, but evidence of focal myocyte necrosis and inflammatory cell infiltration (Figure 3a-c). Spore morphology, as demonstrated by periodic acid-Schiff (PAS) and Grocott’s methenamine silver (GMS) staining, was characteristic of fungi. The muscle specimen was inoculated on medium and the culture grew *C. krusei*, confirming the histopathological diagnosis. Anti-fungal susceptibility tests found sensitivities similar to that of the blood culture. Consequently, intravenous itraconazole was substituted with voriconazole (200mg twice daily) for seven days (days 28 to 34), as antibiotics were gradually discontinued. However, the pyrexia persisted and the lesion had not diminished in size. Our patient declined the offer of excision, so intravenous itraconazole was initiated once again and the decision was made to inject itraconazole 5mg daily into the lesion. Her fever gradually subsided within 48 hours of commencing intra-lesional itraconazole. Then, 10 days later, a further ultrasound scan demonstrated almost complete resolution of the lesion. Once the neutropenia had resolved, the intravenous and intra-lesional itraconazole were discontinued in favor of oral maintenance therapy with itraconazole 200mg twice daily. A follow-up ultrasound scan one month later showed complete resolution of the muscle lesion.

## Discussion

IFI is a recognized consequence of an immunocompromised state, but there are many possible underlying causes. These include neutropenia, exposure to high-dose corticosteroids, the presence of intravascular catheters and prolonged exposure to broad-spectrum antibiotics. Fludarabine also impairs cell-mediated immunity and can contribute to the initiation of IFIs. To the best of our knowledge this is the first case report of a fungal myositis arising in a patient who was neutropenic with acute myelomonocytic leukemia during fludarabine-based chemotherapy in China. Most infectious myositis is bacterial in etiology, with *Staphylococcus aureus* being the most common causative organism. Fungal myositis, although rare, has been described and is a recognized complication of systemic mycosis [2,6-8]. The *Candida* species most often implicated in fungal myositis include *Candida tropicalis*, *C. krusei* and *Candida albicans*[6-8]. The potential routes of infection are generally considered to be hematogenous dissemination, iatrogenic or traumatic with direct inoculation of spores. In our patient’s case, as they had no history of trauma or long-term indwelling catheters, the infection is most likely to have resulted from hematogenous dissemination.

Apart from the treatment of aspergilloma in fibrocavitary lung disease with intra-cavitary amphotericin B [9-11] and *Candida* joint infection with intra-articular amphotericin B [12,13], the local administration of anti-fungal drugs for IFIs is uncommon. Itraconazole was registered for use in 1991 and is widely used to treat several forms of mycosis; however, to the best of our knowledge this is the first case report of the use of intra-lesional itraconazole for fungal myositis.

Pharmacokinetic studies have shown that itraconazole is extensively taken up by the tissues in normal adults and therapeutic levels of itraconazole are maintained many times higher in some infected tissues than plasma. Regardless, in our patient’s case she remained febrile and the myositis persisted despite 22 days of intravenous itraconazole and voriconazole. It is not clear why therapeutic levels sufficient to treat the myositis successfully were not attained in our patient’s case, although it may be that surrounding ischemic and necrotic muscle reduced tissue penetration. We believe that in our patient’s case the systemic treatment alone was inadequate and hypothesize that the use of the combination of intravenous itraconazole and low-dose intra-lesional itraconazole accounted for our patient’s clinical improvement. However, the influence of low-dose intra-lesional itraconazole in the successful outcome of our patient’s case is not completely clear. The clinical response observed could also have been the result of prolonged administration of intravenous itraconazole in the context of recovering neutropenia and the use of oral maintenance itraconazole therapy. No side effects were observed as a result of the intra-lesional administration of itraconazole.

Our patient’s case shows that intra-lesional treatment of fungal myositis with itraconazole is safe and feasible. Future trials should investigate the optimum intra-lesional dose, toxicity and the local application of itraconazole to other tissues.

## Conclusions

We report what is, to the best of our knowledge, the first case of fungal myositis successfully treated with intravenous and intra-lesional itraconazole in a patient with acute myelomonocytic leukemia. The efficacy and safety of the local administration of itraconazole for infected soft tissues requires further evaluation.

## Consent

Written informed consent was obtained from the patient for publication of this case report and any accompanying images. A copy of the written consent is available for review by the Editor-in-Chief of this journal.

## Abbreviations

FLAG: Fludarabine and cytarabine and granulocyte-macrophage colony-stimulating factor; IFI: Invasive fungal infection; M4: Acute myelomonocytic leukemia.

## Competing interests

The authors declare that they have no competing interests.

## Authors’ contributions

X-JL reviewed our patient’s clinical data, performed the literature search and wrote the initial draft. R-XY, M-QH, B-LZ and W-JG reviewed the initial draft and finalized the manuscript. All authors read and approved the final manuscript.
